# Sero-epidemiology study of leptospirosis in febrile patients from Terai region of Nepal

**DOI:** 10.1186/s12879-017-2733-x

**Published:** 2017-09-18

**Authors:** Lalmani Regmi, Kishor Pandey, Meena Malla, Santosh Khanal, Basu Dev Pandey

**Affiliations:** 1 National College, Kathmandu, Nepal; 2 Everest International Clinic and Research Center, Kathmandu, Nepal; 3 Unit of Molecular Biotechnology, Nepal Academy of Science and Technology (NAST), Khumaltar, Lalitpur, Nepal; 4 Department of Health Services, Ministry of Health, Government of Nepal, Kathmandu, Nepal

**Keywords:** ELISA, *Leptospira*, Nepal

## Abstract

**Background:**

Leptospirosis is a re-emerging zoonotic disease caused by pathogenic strains of bacteria belonging to genus *Leptospira* whose symptoms can range from mild clinical manifestations to a severe life threatening illness. This disease may be under-recognized in resource poor settings like Nepal where many clinical laboratories lack appropriate equipment, technology and personnel for proper diagnosis.

**Methods:**

We used IgM ELISA to estimate the sero-prevalence of leptospirosis in a group of febrile patients in a western region of Nepal. We also tested for possible co-infection with two other common febrile diseases endemic to Nepal including dengue and typhoid fever.

**Results:**

Among samples from 144 febrile patients, 30 (21%) were positive for leptospiral IgM. In univariate analysis, leptospirosis was significantly associated with being of working age (*p* = 0.019), farming (*p* = 0.045) and water and animal contact (*p* = 0.0001). Widal and dengue serological study showed that the majority of leptospirosis infections did not have an alternative diagnosis.

**Conclusion:**

As indicated by the study, regular surveillance of animal reservoirs in collaboration with veterinary department and inclusion of leptospirosis as a differential diagnosis of febrile illness is thus recommended based on the current findings.

## Background

Leptospirosis is a zoonotic disease which occurs in diverse epidemiological conditions. It mainly affects vulnerable populations including farmers from rural areas and slum dwellers from urban areas [1]. The outcome of the disease ranges from an undifferentiated febrile illness to life-threatening manifestations such as Weil’s disease and severe pulmonary haemorrhage syndrome. The severe forms of the disease cause mortality in about 5–40% [2]. Therefore, prompt diagnosis and early administration of appropriate antibiotics are important [2]. A recent study estimated that there are annually 1.03 million cases (95% CI 434,000–1,750,000) and 58,900 deaths (95% CI 23,800–95,900) due to leptospirosis worldwide [1]. The estimated 1.03 million cases annually result in a total of approximately 2.90 million Disability Adjusted Life Years (DALY) [3]. Pathogenic *Leptospira* are wide spread and they are capable of surviving in both environment and renal tubule of the animals that get infected [4]. Infection occurs during exposure to animal reservoirs or an environment contaminated by their urine [2]. Many wild and domestic animals are potential reservoirs of the causative spirochetes [5]. These include rodents (especially rats), dogs, cattle, pigs, and horses [6].

Leptospirosis is difficult to diagnose because of its non-specific symptoms, clinical resemblance to other common disorders, and multi-organ involvement [7]. Antibodies are detectable in the blood approximately 5 to 7 days after the onset of symptoms and most cases of leptospirosis are diagnosed by serology [8]. Most commonly used serological tests are Microscopic Agglutination Test (MAT) and ELISA [9]. MAT, however, is slow, tedious, potentially bio-hazardous, subjective and painstaking, requiring the meticulous curating of a collection of strains used alive as antigens [10] and thus is restricted to laboratories that are capable for maintaining strains for preparation of live antigens [11]. The immunoglobulin M (IgM) enzyme-linked immunosorbent assay (ELISA) is one of the alternatives to MAT [12]. ELISA is not serovar specific but is an excellent choice in screening and surveillance [10].

There is a high prevalence of leptospirosis in Asian countries. Frequent outbreaks occur in developing countries related to overcrowding, poor sanitation, and climactic condition [13]. Epidemics of leptospirosis have been reported in Sri Lanka in 2008 and the Philippines in 2009 [14]. Most cases reported from India are from the four states of Kerala, Gujarat, Tamil Nadu and Maharashtra [15, 16]. The first report of a suspected leptospiral infection in Nepal was in a Nepali soldier in 1981 [17]. Since then, a number of serological studies have been carried out in Nepal, showing the presence of antibodies against *Leptospira* in a number of populations [18–21]. Leptospirosis is an under diagnosed disease in Nepal and easily mistaken for other febrile illnesses. This study was undertaken to evaluate the seroprevalence of leptospirosis in the Terai region of Nepal and recommend the health practitioners to consider the disease in differential diagnosis of febrile illness.

## Methods

### Study population

**Fig. 1:**
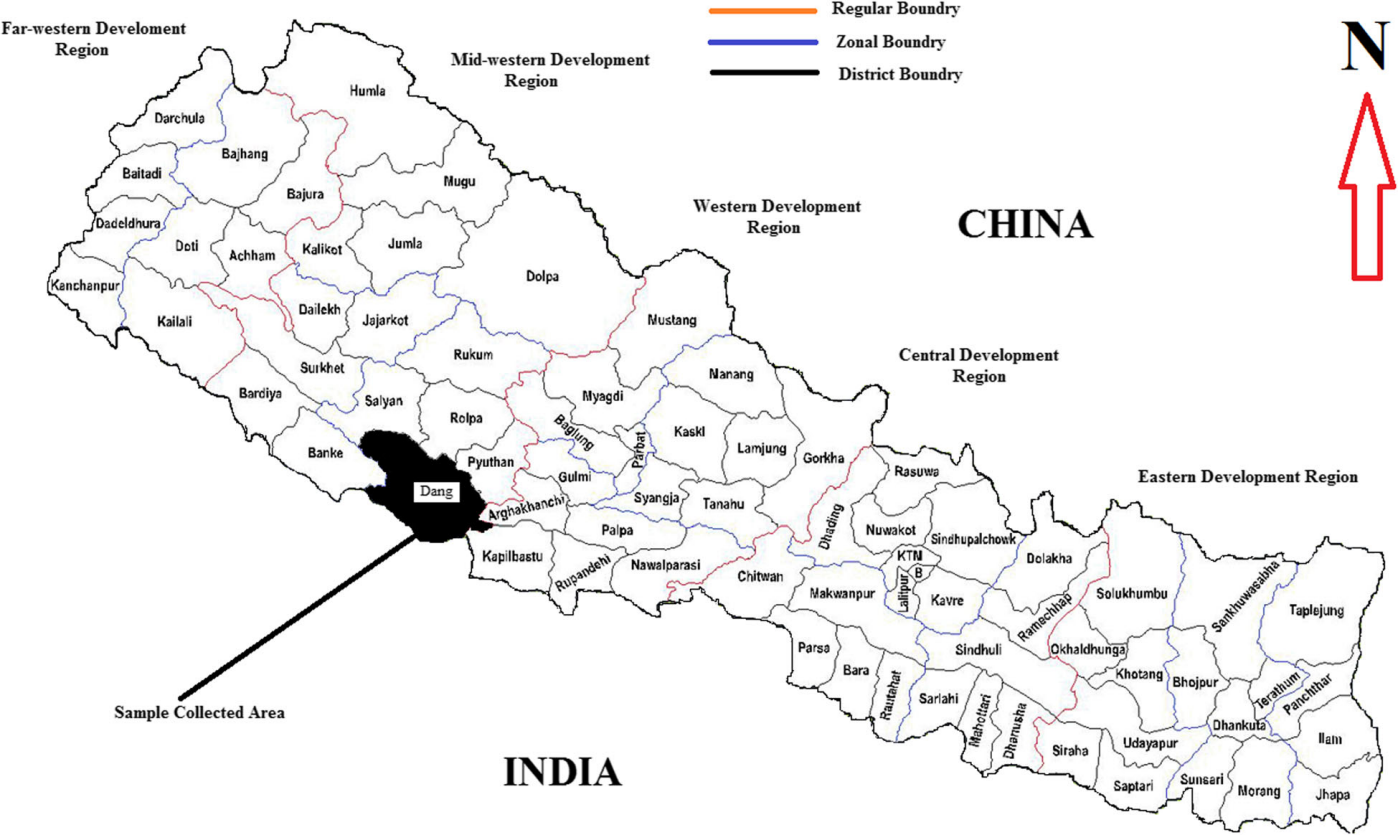
Map of Nepal showing sites of serum sample collected district (Source: www.marketwatch.footprints.com.np)

A total of 144 febrile patients who visited a government hospital (Rapti Zonal Hospital) and three private clinics (Dirghayu Polyclinic, Palpali Polyclinic and Kamana Pharmacy) in Dang, Western part of Nepal were included in the present study (Fig. 1). The study was carried out in the monsoon period of June–August 2014. A blood sample was collected from all febrile patients at the time of their hospital/clinics visit. We collected blood samples from the febrile patients who had fever at least 3 days. We excluded the patients’ with fever for less than 3 days. Serum samples exhibiting haemolysis, lipaemia or microbial growth were also excluded from the study [22]. Samples were centrifuged, stored, and shipped at 4 °C to the Everest International Clinic and Research Centre, Kathmandu. A standardized questionnaire was used to collect patients information regarding demographic details (age, sex, and occupation), clinical symptoms (fever, headache, myalgia, vomiting, diarrhea, and abdominal pain), animal or water contact, and presence of pets at home.

### Serological study

Detection of IgM antibodies to *Leptospira* was determined using a commercially available *Leptospira* IgM capture using a microtiter plate ELISA (Panbio Leptospira IgM ELISA, Queensland, Australia). The ELISA test was performed according to the manufacturer’s protocol and interpreted either positive or negative on the basis of absorbance with respect to cutoff values. The sensitivity and specificity of the ELISA kits were 96.5% (95% CI = 87.9–99.6%) and 98.5% (95% CI = 95.6–99.7%). A sample was considered “Positive” if Panbio units calculated for it is more than 11 IU/mL. A sample was considered “Equivocal” if Panbio units calculated for it is 9–11 IU/mL. A sample was considered “Negative” if Panbio units calculated for it is less than 9 IU/mL. Briefly, test sera, cutoff calibrator, and positive and negative control sera were diluted 1:100 in serum diluents, and 100 μL added to *Leptospira* antigen-coated microwells and incubated for 30 min at 37 °C. After washing with phosphate-buffered saline containing 0.05% Tween 20, 100 μL of HRP conjugated anti-human IgM was added and incubated for another 30 min at 37 °C. After further washing, 100 μL of tetramethylbenzidine substrate was added and incubated at room temperature for 10 min, after which the reaction was stopped with 100 μL of 1 M phosphoric acid. The absorbance of each well was read at a wavelength of 450 nm with a Bio-Tek ELX 808 plate reader (Bio-Tek Instruments, Winooski, VT). The widal test was performed to detect antibodies against O and H antigens. The samples which showed agglutination with the antigenic reagents were considered positive and those that do not agglutinate were considered negative. Detection of anti-dengue IgM by ELISA was performed using SD dengue IgM capture ELISA (Standard Diagnostics Inc.) according to the manufacturer’s instructions.

### Statistical methods

Statistical package for social science (SPSS) software (version 17.0) was used for data entry and analysis. Association of different variables to leptospirosis was analyzed by using Chi-square test and *p* < 0.05 was considered significant.

## Results

Most of the patients were adults with a mean age of 31.75 years. The majority of *Leptospira* IgM positive cases (43.3%) were from the 31–40 years age group followed by the 21–30 years age groups with 29.3% positive for leptospirosis. The youngest age showing positive result was 12 years and the oldest age was 58 years. Among the Leptospira IgM positives, most were adults with mean age of 28.3 years. Statistically there was significant association in the presence of leptospirosis among various age groups (*p* = 0.019) (Table 1). The male and female ratio is 1: 1.1. Out of 68 females, 17 (25.0%) were found to be serologically positive. Similarly, 13 (17.1%) out of 76 males were found to be serologically positive for leptospirosis. Statistically there was no significant difference in the presence of leptospirosis among sexes (*p* = 0.244) (Table 1).

**Table 1:** Demographic presentation of leptospirosis

Age of patients	ELISA for febrile patients		*P*-value
	Positive (%)	Negative (%)	
<10	0 (0.0%)	9 (6.3%)	0.019
11–20	6 (4.2%)	24 (16.7%)	
21–30	12 (9.0%)	29 (20.1%)	
31–40	10 (6.3%)	15 (10.4%)	
41–50	0 (0.0%)	15 (10.4%)	
51–60	2 (1.4%)	11 (7.6%)	
61–70	0 (0.0%)	9 (6.3%)	
>70	0 (0.0%)	2 (1.4%)	
Gender			
Male	13 (9.0%)	63 (43.8%)	0.244
Female	17 (11.8%)	51 (35.4%)	
Total	30 (20.8%)	114 (79.2%)	

Of the 22 cases having animal contact, 13.6% were positive for IgM against *Leptospira* species. Similarly of the 32 cases having water contact, 43.8% were IgM positives against *Leptospira*. Among the cases having animal and water contact, 39.1% were positive for IgM ELISA. Of the cases that had animal or water contact, 5.9% were positive for leptospirosis by ELISA. Statistically significant association was found between animal/water contact pattern and leptospirosis infection (*p* = 0.0001) (Table 2).

**Table 2:** Risk factor association with leptospirosis

Risk factors	ELISA for febrile patients		*P*-value
	Positive (%)	Negative (%)	
Animal contact	3 (2.1%)	19 (13.2%)	0.001
Water contact	14 (9.7%)	18 (12.5%)	
Both	9 (6.3%)	14 (9.7%)	
Other	4 (2.8%)	63 (43.8%)	
Total	30 (20.8%)	114 (79.2%)	

The most common clinical symptoms shown by the 144 patients were fever (100%), headache (69.4%), Myalgia (55.4%), vomiting (13.9%), diarrhea (13.9%), abdominal pain (13.9%) and Jaundice (8.3%) as shown in Table 3. Patients were subjected to routine laboratory checks, including white blood cells (WBCs), platelets, and hemoglobin (Hb) levels. Out of 144 serum samples from patients suspected of leptospirosis, 30 (20.83%) were positive for anti-*Leptospira* IgM antibody. Clinical sign and symptoms of the 30 ELISA positive cases of leptospirosis were further analyzed. Fever, headache and myalgia were the most common symptoms of ELISA positive cases. The present study also revealed leptospirosis to be most common among farmers (26.7%), followed by students (23.3%), masons (20%), housewives (13.3%), servicemen (10%) and businessmen (6.7%). Of the 30 sero-positive patients, two were involved in business and three in the service sector (data not shown).

**Table 3:** Clinical presentation of the patients visited to hospital/clinics

Clinical Sign/Symptoms	No. of cases showing symptoms (%)	Percentage of ELISA positive cases (%)
Fever	144 (100)	30 (100)
Headache	100 (69.4)	23 (76.3)
Myalgia	80 (55.4)	21 (70.1)
Vomiting	20 (13.9)	9 (30.5)
Diarrhoea	20 (13.9)	8 (26.4)
Abdominal pain	20 (13.9)	5 (16.7)
Jaundice	12 (8.3)	4 (13.9)
	Value	
Hb, g/dL (*N* = 144)		
Median	12.5	
Minimum	7.5	
Maximum	19.8	
WBC, per μL (N = 144)		
Median	8500	
Minimum	1100	
Maximum	32,400	
Platelets, per μL(N = 144)		
Median	176,000	
Minimum	16,500	
Maximum	4,000,001	

Only one of the patient’s positive for leptospiral IgM ELISA was also positive for the Widal. Similarly, only one patient positive for leptospiral IgM ELISA also tested positive for dengue IgM.

## Discussion

We show that a large proportion (21%) of febrile patients presenting to a Nepali hospital/clinics during summer season had serologic evidence of acute leptospirosis. A study conducted at Chitwan Medical College across 1266 patients suspected of leptospirosis revealed sero-positivity in 61 (4.8%) cases [21]. When restricted to analysis of patients presenting during the summer, their sero-positivity rate was 21.3%, which is in line with our findings.

Most of the sero-positive cases were within the 21–30 and 31–40 age groups. The clustering of the disease in these age groups is consistent with occupational exposures in these “working” ages. In the study carried out by Seti et al., [23] most of the patients (70%) positive for leptospirosis were young adults in their 2nd, 3rd or 4th decades of life. Similarly, in another study done by Chawla et al. [24], the highest percentage of positive for leptospirosis cases were from the middle age group. We found a higher incidence of leptospirosis among females that was not statistically significant, in contrast with previous studies demonstrating higher incidence of leptospirosis in males [23–26]. The higher incidence in females at our site may be due to emigration of males to foreign countries for work, resulting in an increase in female participation in farming and other work previously dominated by males.

Of the cases having animal contact (cattle, buffaloes and pets), 26.6% were seropositive. Rearing cows and/or buffaloes is a common practice in Nepal, and these animals are often chronically colonized by pathogenic *Leptospira* with frequent transmission to humans [27]. The Directorate of Animal Health, Nepal reported an overall 10.5% incidence of leptospirosis in cattle and buffalo, goat, sheep and pig. Dogs have become popular pets with a risk of *Leptospira* transmission [28]. In a study conducted in 150 dogs presenting with fever and jaundice, serological positivity for leptospirosis was established in 2.7% (4/150) of those [29]. Similarly, of the cases having water contact, 41.8% were ELISA positive. The study period coincides with the paddy growing season in Nepal, and an increase in leptospirosis has been associated with the rice paddy harvesting season where an increase in the rodent population in and around the field is observed [26]. Swimming in pools or white water rafting as a recreational activity is becoming popular in urban areas of Nepal too. Numerous studies have shown that such activities have been associated with leptospirosis [30, 31].

Leptospirosis has traditionally been considered a disease of farmers [29, 30]. In accordance to these findings, the present study also revealed to be most common among farmers, followed by students, masons, housewives, servicemen and businessmen. Their profession didn’t carry any risk for leptospirosis and seems like they might have acquired it by accidental animal/water contact.

One of the patients who tested sero-positive for leptospirosis also tested positive for Widal test. Clinical manifestations did not hint at the worsening of clinical symptoms of the patient, as expected for the co-infections. Blood samples from the patient in the first week of fever was not available for culture to trace the microbial etiology of the Widal positivity (convalescence samples were also not available). Since this test is non-specific, positive results may also indicate the cross reactivity with antibodies specific to bacterial (members of enterobacteriaceae) and non-bacterial (malaria, dengue, hepatitis A, and infectious mononucleosis) diseases and may have previous infection [32].

1/144 samples also showed seropositivity for both leptospirosis and dengue specific IgM. The positive IgM result for dengue could be considered an old infection. The IgM antibodies for dengue generally remain in circulation for prolonged periods of time. Clinical presentation of dengue fever and leptospirosis are considerably overlapping, leading to misdiagnosis in cases of mixed infection. In acute stage of infection, both present as acute febrile illness with chills, myalgia, headache, abdominal pain, and anorexia. Though dengue IgM can persist for months, the severity of the observed symptoms in the patient suggested that it could more likely be a simultaneous infection of dengue and leptospira. This patient also presented arthalgia in addition to non-specific symptoms of leptospirosis, inferring the symptomatic aggravation by the co-infection. Such co-infections have been previously reported in Nepal and India [33, 34].

This study has several limitations, the greatest of which is its cross-sectional nature, as we were not able to collected paired serum samples. Secondly, we only collected samples over a short duration, though we chose the summer season as it has the highest incidence of disease. Thirdly, MAT, the gold standard test for leptospirosis could not be done, nor we could perform blood culture or any molecular tests. However, we believe that the result of this study describes the general features of the disease in Nepal.

## Conclusion

This study indicates Leptospirosis is a significant public health problem in Nepal. In resource poor countries like Nepal where laboratories performing MAT or maintaining cultures are rarely available, serological test like ELISA could well depict the scenario of the disease prevalence. This study highlights urgency of comprehensive investigation in coordination with animal health department as a one of the health approaches to find the situation of leptospirosis in Nepal and accordingly to formulate reflect in policy for control of disease.

## Abbreviations

DALY: Disability adjusted life years
ELISA: Enzyme-linked immunosorbent assay
IgM: Immunoglobulin M
MAT: Microscopic agglutination test
